# Supplementary material for: State-dependent brainstem ensemble dynamics and their interactions with hippocampus across sleep states
Source: eLife. 2020 Jan 14;9:e52244. doi: 10.7554/eLife.52244 (PMC6996931; doi:10.7554/eLife.52244)
Supplement: Supplementary file 1. — BS-Si, silicon probe recording in the brainstem. HP-Si, silicon probe recording in the hippocampus. BS-EEG, EEG recording in the brainstem. FP, fiber photometry. Exc1, the animal was excluded because of electrode mispositioning. Exc2, the animal was excluded because of lack of histological data. Exc3, the animal was excluded because of eye closure during recording. [file elife-52244-supp1.docx]

**Supplementary file 1. Summary of experimental animals and recordings in this study.**

BS-Si, silicon probe recording in the brainstem. HP-Si, silicon probe recording in the hippocampus. BS-EEG, EEG recording in the brainstem. FP, fiber photometry. Exc^1^, the animal was excluded due to electrode mispositioning. Exc^2^, the animal was excluded due to lack of histological data. Exc^3^, the animal was excluded due to eye closure during recording.
